# From MRIcro to MRIcron: The evolution of neuroimaging visualization tools

**DOI:** 10.1016/j.neuropsychologia.2025.109067

**Published:** 2025-01-04

**Authors:** Christopher Rorden

**Affiliations:** McCausland Center for Brain Imaging, Department of Psychology, University of South Carolina, Columbia, SC, 29016, USA

## Abstract

Visualization software is a critical component at every stage of neuroimaging research. It enables researchers to inspect raw or processed datasets for artifacts, to identify anomalies, to verify the accuracy of automated processing, and to interpret the location of statistical results within the complex structure of the human brain. Since 2006, MRIcron has provided a free, open-source, cross-platform tool designed to meet these needs. Despite its minimal system requirements, MRIcron supports various popular neuroimaging file formats, ensuring compatibility with widely-used tools in the field, such as SPM, FreeSurfer, FSL, and AFNI.

The intuitive graphical interface allows for straightforward image visualization and manipulation, while its advanced features such as lesion drawing and ability to handle many image formats cater to more sophisticated analyses. Furthermore, MRIcron’s scripting capabilities enable users to automate complex workflows, facilitating the efficient processing of large datasets.

In summary, MRIcron is a powerful and versatile tool that addresses the visualization and analysis needs of the neuroimaging community, contributing to the advancement of brain research by providing a reliable and efficient solution for brain imaging analysis. This article describes the development of MRIcron, from its inception to the present day.

## 1. Introduction and background

Neuroimaging research relies heavily on effective visualization tools at every stage of analysis. Such tools are essential for inspecting raw images for artifacts, identifying anomalies, verifying the accuracy of automated processes, and interpreting the location of statistical results within the complex structure of the human brain. MRIcron is a free, open-source (https://github.com/neurolabusc/MRIcron), and cross-platform software specifically designed to address these needs. We describe the origins of this software, the design goals, implementation, features, and limitations.

It is fitting to describe this tool in a special issue of Neuropsychologia focused on celebrating John Duncan’s career. The software fulfills one of his visions, and his efforts facilitated its development. One of the most remarkable aspects of John Duncan’s work is his visionary approach and skill in utilizing a wide range of methods to explore brain function. Unlike the traditional model of a successful scientist, which often emphasizes mastering a single technique, John excels in integrating complementary tools to address core questions, usually centered around a refined behavioral paradigm. This approach is particularly impressive given the state of these tools when he began his research—many were in their early stages of development. John not only employed techniques like electrophysiology, functional brain imaging, and lesion mapping, but also contributed to building the infrastructure and developing the analyses needed to fully harness these emerging methods. His work can be likened to a well-crafted detective novel: while individual pieces of evidence might seem circumstantial, together they form a compelling and insightful narrative.

I leave it to the other articles in this issue to highlight the benefits of various methods that John championed. John’s work focused my career around the single method of using human brain imaging to gain insight into the architecture of the brain. In particular, I do think we can learn a tremendous amount by using brain imaging to understand the consequences of and recovery from brain injury (Moore et al., 2024). This endeavor has clear clinical implications - providing a prognosis (Bonilha et al., 2016), identifying risk factors (Roth et al., 2023), and guiding the personalized treatment of precision medicine (Fridriksson et al., 2018). Brain imaging, initial symptom severity, and demographic factors can be combined synergistically to predict recovery trajectories (Busby et al., 2023). These baseline predictions can improve the statistical power of interventional trials by balancing individuals across treatment arms based on similar projected outcomes. However, it can also provide compelling information regarding the operation of the healthy human brain. While measures of brain activation identify regions involved in a task, brain disruption provides a stronger inference - identifying the regions required for a task. Ironically, my single-minded pursuit of this method was set into motion by John Duncan, one of the most profound polymaths I know.

In the United States, ‘My Big Break’ is a popular series on the National Public Radio series. It chronicles the often random ‘life-changing moments when people leap forward in their careers’. Personally, I often think of my post-doctoral years with John Duncan and Jon Driver as my own big break. Commencing in 1996, their Wellcome Trust supports project envisioned creating a large database of stroke survivors, combining acute hospital MRI imaging data with a chronic measure of visual perception (Peers et al., 2005). Our team needed to devise methods to map the extent of brain injury and warp the locations from different individuals into a common stereotaxic space (Brett et al., 2001). When I started work on this project, we used the professional Analyze (Robb et al., 1990) software, which only ran on SUN workstations. Likewise, the seminal free BrainVox (Frank et al., 1997) required expensive Silicon Graphics workstations that we did not have. Due to the cost of the hardware and software, we only had a single Analyze license for the entire institution. This meant that different users needed to coordinate usage. Dr Nagui Antoun was initially tasked with drawing the lesion maps, and was unimpressed with these tools, telling me he just wanted something that could run on his ‘micro’ (at the time, ‘microcomputer’ was a common term for a personal computer, in contrast to the expensive scientific workstations).

Therefore, one Friday I told John my idea of creating ‘MRIcro’ - a tool for visualizing MRI scans on a microcomputer. To this day, John accurately retells the story of how he told me this would be a waste of my time, so I spent all weekend creating a mock-up for him to see the following Monday. However, it is worth noting that after this John was a strong supporter, as it fit into his grand vision for developing the infrastructure for lesion mapping. At this stage, credit must also be given to Matthew Brett, who conceived many important new features and roles for the software. While MRIcro was designed for a single radiologist to use, it proved popular as a free download that could run on any Windows computer. Indeed, Google Scholar notes that the manuscript describing MRIcro (Rorden and Brett, 2000) has been cited more than 2800 times.

However, the minimal design of MRIcro had severe limitations. First, we initially needed a tool that could display 16 mb MRI scans on a computer with just 20 mb of RAM. This led to a lot of compromises. For example, MRIcro used a 256-level 8-bit color palette with 255 levels of gray for the MRI scan, while reserving one opaque color (red by default) for the brain injury map. This precluded the ability to have transparent lesions. Within a few years, we had computers with far more memory, but the original software was inherently designed to run lean.

Likewise, MRIcro was created on computers with poor floating-point performance, so it relied on integer calculations that traded precision for speed. In addition, while the core motivation was to create software that could run on commodity Windows microcomputers, it was created at a time when users were quickly adopting Linux and Macintosh computers. Another inherent limitation of MRIcro is that it was designed to view Analyze format images, which lacked spatial transformation information. Indeed, different popular neuroimaging tools typically used their own formats. The introduction of the NIfTI format (Cox et al.) provided a unified interchange of volumetric datasets, as well as spatial transformations in its header that MRIcro did not support.

The original rationale for creating MRIcro was to have a tool to map lesions. These lesion maps aid the spatial normalization of each individual’s image into a standard space (Brett et al., 2001) and the subsequent voxel-based lesion symptom mapping statistics (Bates et al., 2003). Therefore, MRIcro did provide a simple pen tool for drawing binary (a voxel could either be lesioned or unlesioned) lesion maps. To reduce disk storage requirements, these lesion maps were stored using a very simple compression (known as run length encoding). While fast and efficient, this format had severe limitations. This format was not support by other tools, and was limited to images with a maximum of 65536 pixels in-plane. Even at the time of creation, some high-resolution 2D clinical images exceeded this limit.

In sum, while MRIcro proved popular for its specific niche, it had inherent limitations that constrained its broader impact. These limitations, stemming from the early technological constraints and the evolving landscape of neuroimaging tools, necessitated the development of a more robust and versatile solution. Thus, MRIcron (MRIcro for NIfTI) was conceived as a clean-sheet redesign to overcome these challenges, offering improved functionality, compatibility, and performance. In the following section, we will detail the design and implementation of MRIcron, highlighting the key features that address the limitations of its predecessor, as well as the novel capabilities that position MRIcron as a powerful tool for the neuroimaging community. This discussion will also include an evaluation of MRIcron’s performance across different platforms and its integration with other leading neuroimaging tools, demonstrating how it meets the current needs of researchers and clinicians in the field.

## 2. Design and implementation of MRIcron

As noted, MRIcron was conceived to address the inherent weaknesses of MRIcro. In particular, the ability to leverage contemporary hardware, cross-platform support for different operating systems and the ability to seamlessly support image formats such as NIfTI.

A major goal of MRIcron was to provide a cross-platform tool with the same functions regardless of whether the user prefers the Linux, Macintosh or Windows operating system. While it is relatively easy to create cross-platform command line programs, creating cross-platform graphical desktop applications requires compromises. A popular solution for this problem is to use the QT software library. For example, FSLview and FSLeyes from the FSL team 13, 3D Slicer 14, and FreeSurfer’s FreeView (Dale et al., 1999) each use the QT library (though note that only 3D slicer support Windows). In contrast, AFNI (Saad et al., 2004) uses the Motif library to support Linux and MacOS (but not Windows). Another option for cross-platform visualization is to use the proprietary Matlab language, as seen with SPM (Friston et al., 1994). All of these options use their own graphical display elements such as windows, menus and buttons, which adds overhead and means that they do not feel native. For these reasons, MRIcron took an unusual route, using the Pascal language and the Lazarus cross-platform integrated development environment. Indeed, beyond supports multiple operating systems, this environment can also target multiple architectures, for example our MacOS version of MRIcron runs natively on different types of processors, including PowerPC, Intel ×86 and ARM architectures. This provides a write-once, run anywhere solution that uses the native graphical elements of each operating system. This results in an executable that requires little disk space, has few dependencies, and looks and feels like other tools. Despite these advantages, choosing an unpopular programming language has meant that all updates and enhancements have been carried out by a single individual.

**Fig. 1.**
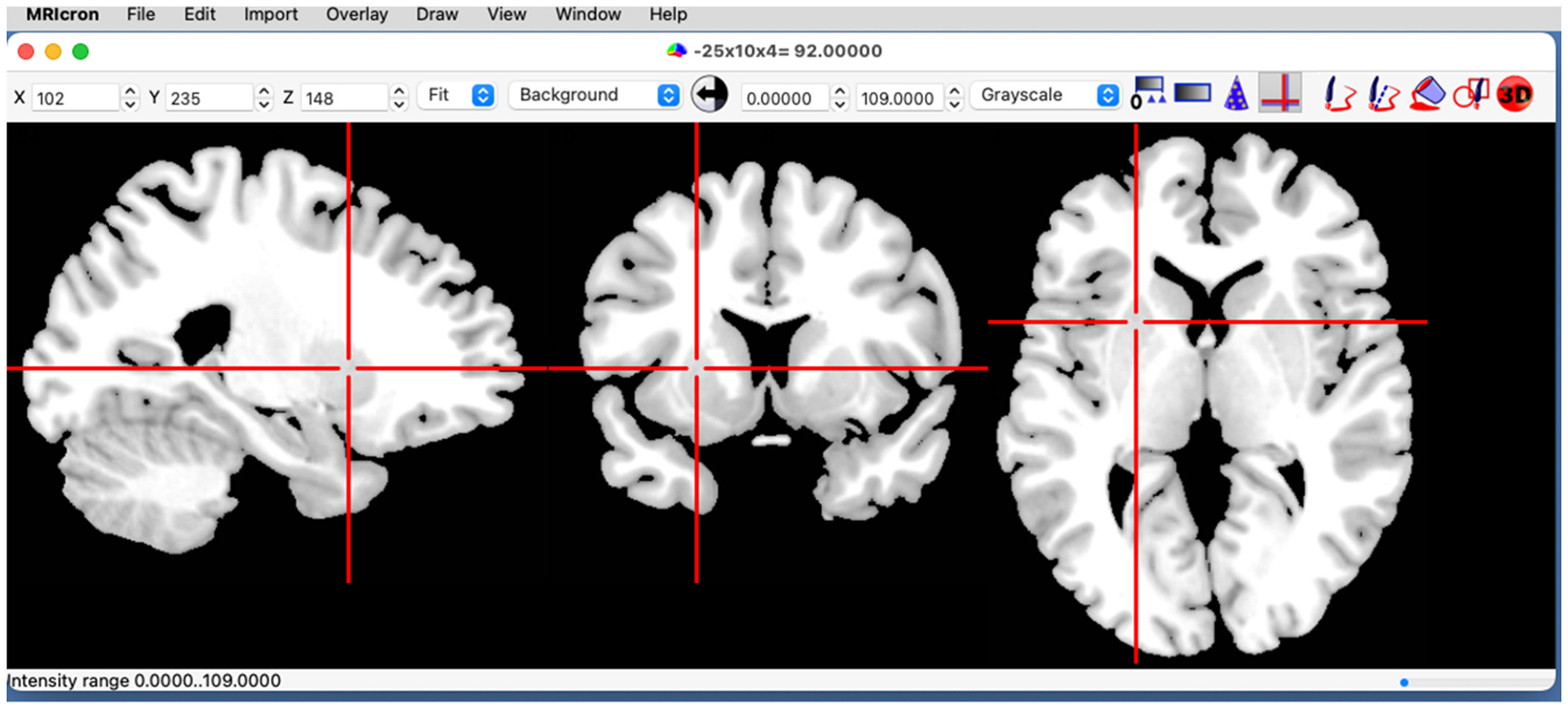
The MRIcron user interface. The main menu provides access to core functions. Users can change the crosshair position by clicking on the image or setting the coordinates in the top toolbar, allowing users to specify the crosshair coordinates (with X, Y, Z referring to the column, row and slice of the image). Note that the title bar reports the crosshair location in world space as well as the voxel intensity. Likewise, users can set contrast and brightness by dragging over the image or using the minimum and maximum thresholds in the titlebar. A pull-down menu allows the user to select the color scheme (here Grayscale). Buttons in the toolbar allow the user to display a gradient colorbar and draw lesions. The bottom status bar reports recent changes (here the new contrast values).

MRIcron uses the Windows, Icons, Mouse, Pull-down menus (WIMP) user interface, common with contemporary desktop applications (Fig. 1). Users can install MRIcron by downloading the latest version from the NeuroImaging Tools & Resources Collaboratory (https://www.nitrc.org/projects/mricron/). This design allows users to interactively discover commands. A user manual is also provided online (https://www.nitrc.org/plugins/mwiki/index.php/mricron:MainPage). Users can seek help from the online forum (https://www.nitrc.org/forum/?group_id=152). The source code is available from Github (https://github.com/neurolabusc/MRIcron).

**Fig. 2.**
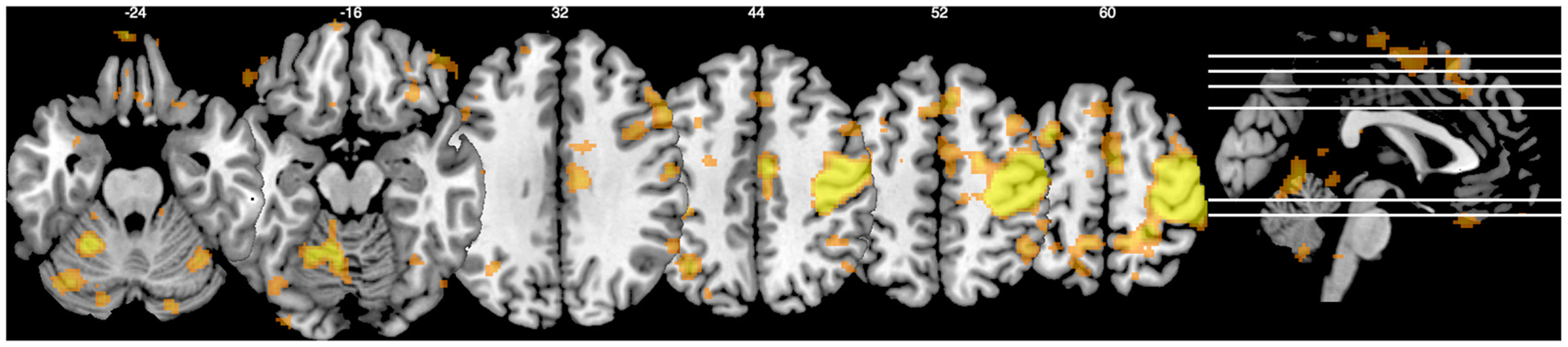
MRIcron is able to create mosaic views that show multiple slices simultaneously. Note that the user can arbitrarily select which slices to show (i.e. in this example the slices are not equidistant). This figure also shows the translucent blending of overlay images, and option to have partial overlap across slices.

The development of MRIcro was shaped by the limitations of the hardware available at the time, requiring compromises to accommodate these constraints. In contrast, MRIcron leveraged the relatively more capable hardware available at the time of its development, while still aiming to work with the commodity hardware of that period. Specifically, MRIcro was compiled as a 32-bit executable, while MRIcron targeted 64-bit hardware. In addition to extended memory addressing, all x86–64 computers included relatively powerful floating point capabilities as well as other optimisations such as limited parallel data processing (for example Single Instruction Multiple Data features) and multiple CPU cores. Taking advantage of these features allowed MRIcron to benefit from the increased precision associated with floating point computations, and to include features such as loading multiple images simultaneously. The graphical display also supporting a full (Rorden et al., 2024) bit rather than 8-bit color palette that allows translucent blending of the different layers, as shown in Fig. 2. The Figure also illustrates the signature option for the mosaic views to show successive slices with partial overlap. This effect maximizes the voxel size for Figures when embedded into a journal page, and is particularly useful for studies of unilateral lesions (e.g. aphasia (Riccardi et al., 2023) and spatial neglect (Karnath et al., 2004)), while it is less useful for many functional imaging studies where bilateral activations are common.

**Fig. 3.**
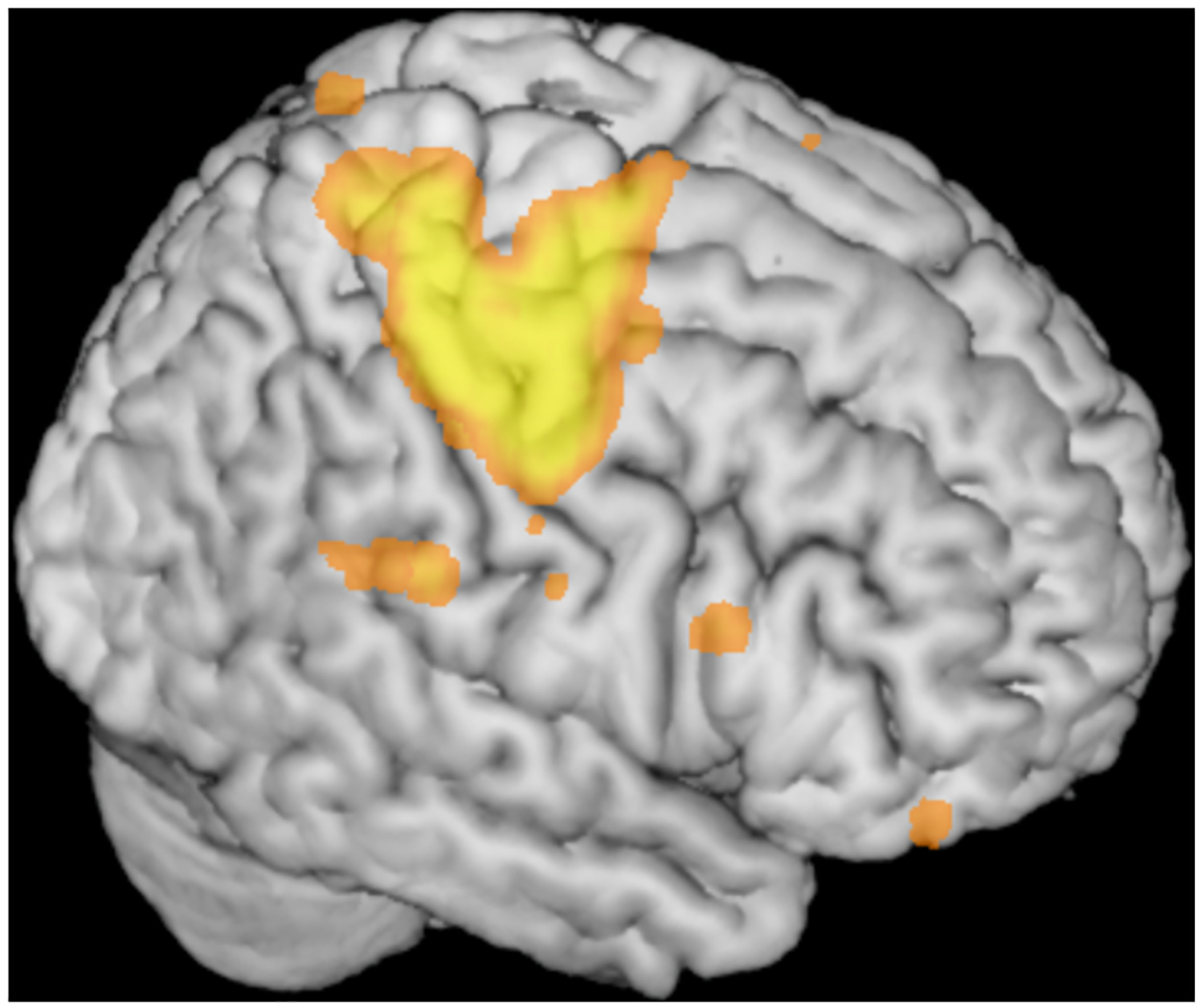
MRIcron provides volume rendering to allow users to visualize the pattern of cortical folds, location of activation and patterns of injury.

MRIcron also provides rudimentary volume rendering (Fig. 3). The rendering uses the central processing unit (CPU), so it does not require any special hardware. A consequence of this is that the 3D rendering is not interactive. This reflects the fact that when MRIcron was written commodity computers did not have the hardware to supported these features (the OpenGL 3.0 software library that introduced 3D textures aiding interactive volume rendering was not released until 2008). While this is a limitation when compared to more recent visualization tools, it does mean that MRIcron is able to run easily on platforms such as neurodesk (Renton et al., 2024) without requiring modern graphics card capabilities.

**Fig. 4.**
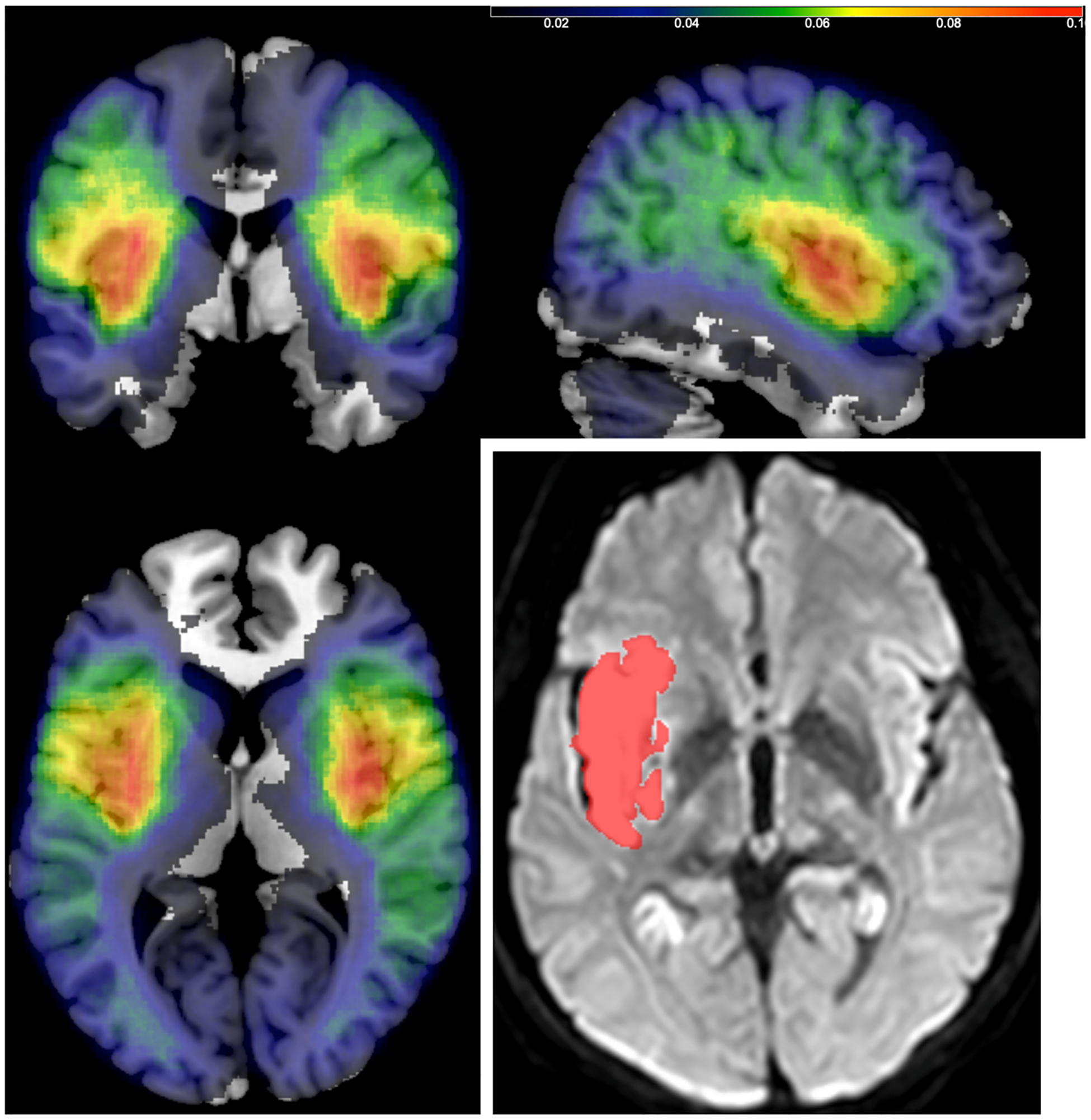
MRIcron introduced improved drawing tools to map lesions on individual participants (inset on right, sub-09) and create lesion incidence maps. The multi-colored incidence map overlay shows the fractional incidence (thresholded for blue at 1% and red at 10%) for 1449 individuals. Both images from the Stroke Outcome Optimization Project (SOOP) Absher et al., 2024.

As previously described, MRIcro’s support for lesion mapping was very primitive. MRIcron was created as the lesion mapping and visualization component for a trio of tools designed to provide an end-to-end pipeline (de Haan and Karnath, 2018) for lesion symptom mapping (Fig. 4). Specifically, users could draw lesions with MRIcron and align each individual’s scan to stereotaxic space with the clinical toolbox (Rorden et al., 2012), and to conduct statistics with our nonparametric mapping tool (Rorden et al., 2009) and visualize the results with MRIcron. MRIcron provides a range of drawing tools, including options to draw and erase lines, fill regions, create circles and ellipses, and use a ‘bucket’ tool for filling enclosed areas. Additionally, it includes basic morphological operations to select regions based on image intensity. Users can copy and paste drawings between slices, making it easy to edit only the portions that change across slices. The lesion maps were stored using the NIfTI format, with the ubiquitous Gzip compression format ensuring minimal disk requirements (by default, MRIcron saves these drawings with the extension ‵.voi‵ to allow easy file selection, but they can be recognized and opened with many tools by using the ‵.nii.gz‵ file extension).

As noted, a major impetus for creating MRIcron was the NIfTI format (Cox et al.) that provided a single unified format for the neuroimaging community. Since its release, NIfTI has become the interchange format for our domain and the core image format for the BIDS data structure (Poldrack, 2024). However, many popular tools still default to their own proprietary formats, in particular for intermediate stages of processing. Observing intermediate images is often a useful way to detect, diagnose and resolve processing problems. Specifically, visual inspection can reveal acquisition errors (e.g. radio frequency artifacts), reconstruction errors, data transmission/storage errors, motion artifacts, spatial coregistration errors, and spatial normalization errors (Taylor et al., 2024). To handle this, MRIcron automatically detects and reads the native image formats of many of the domain’s popular tools. Specifically, it is able to read AFNI (head/brik) (Cox, 1996), BrainVoyager (V16, VMR) (Goebel et al., 2006), Blender (BVOX), FreeSurfer (mgh, mgz) (Dale et al., 1999), ECAT, NRRD, and legacy VTK (vtk) format (Schroeder et al., 2006) images. It also includes a graphical wrapper for dcm2niix (Li et al., 2016) that allows conversion of DICOM images to the NIfTI format.

Visualization is essential for understanding and sharing results. Visualization techniques range along a continuum from precision to interpretability (Pernet and Madan, 2020): 2D slices offer precision, while 3D rendering enhances interpretability, aiding in the recognition of complex structures. MRIcron supports both 2D slices (Fig. 2) and 3D rendering (Fig. 3), as well as hybrid renderings with slice cut-outs to combine these approaches. In all cases, statistical maps can be overlaid on a high-quality anatomical scan, providing recognizable landmarks for clearer interpretation. Again, MRIcron supports the common and proprietary formats common in our domain.

A common problem for any MRI visualization tool is how to represent the discrete voxel data contained in the image on the discrete pixels of a computer screen, as there is rarely a direct one-to-one mapping. Voxel sizes are often anisotropic while screen pixels are square. Images are often acquired with slices aligned to anatomical features rather than the coordinates of the scanner bore, and voxel data may be stored on disk in an arbitrary order and use spatial transforms to warp the data into a desired orientation (e.g. see https://github.com/rordenlab/NIfTIspace). In situations such as lesion tracing where there is a binary mapping of voxels as being either being spared or injured, one may prefer to display data in the original image space (aligned to the image slices as acquired) to avoid aliasing effects. In other situations (such as when comparing data across participants) it may be desirable to display images in a common spatial reference frame (“world space”). For this reason, MRIcron allows the user to choose whether to display volumes in image (square voxels with nearest neighbor interpolation) or world space (reslicing data using trilinear interpolation).

MRIcron is distributed using a permissive free and open source license. This is in contrast to other popular free software in our domain that uses restrictive licenses (Rorden et al., 2024). The license allows others to contribute, evaluate and even commercialize the source code. It can be pre-installed easily and deployed without requiring any special hardware or software keys.

## 3. Discussion and future directions

MRIcron has proved popular, and it is listed as the top download from the NeuroImaging Tools & Resources Collaboratory website (https://www.nitrc.org listing 872655 which does not include other downloads, for example direct from GitHub). Despite this, I have always found it challenging to write peer-review articles describing my software. Indeed, MRIcron was first introduced in 2006, so this manuscript is almost twenty years overdue. The informal preamble explains the situation: I create tools to solve the scientific problems my teams are working on, and the adoption of these tools by other teams is a side effect. Further, my scientific work on perception and aphasia is a more natural fit for the structure of scientific articles. In writing this manuscript, I felt a strong kinship with ImageJ (Schneider et al., 2012) which was also created to bring scientific visualization to low-cost computers, with a scientific manuscript lagging years after its introduction. Therefore, I am grateful for this special issue for providing me with an impetus to describe this tool as well as to recognize the visionary role that John had in developing the infrastructure for the domain I now work in. In retrospect, my early visualization tools arrived at a pivotal time-—offering free, user-friendly ways to visualize MRI data that greatly enhanced accessibility to neuroimaging research methods. Today, the collaborative scientific software community provides a wealth of free tools, with modern hardware enabling developers to prioritize advanced features and maintainability without the constraints that once shaped earlier software for limited hardware.

Like MRIcro before it, MRIcron was designed to leverage the hardware available at the time of its creation. Although websites like NITRC suggest it remains popular, it does not take advantage of recent innovations. Consequently, my development efforts have shifted to its successors, the hardware-accelerated MRIcroGL and the web-based NiiVue.

The direct successor to MRIcron is MRIcroGL. MRIcroGL uses modern graphics hardware to provide interactive volume rendering. It also provides a built-in Python interpreter that allows for scripting of visualizations. These scripts allow new users to explore the software capabilities by choosing from a series of included tutorial scripts. It also allows advanced users to automate arduous tasks and ensure reproducible images.

My most recent visualization tool is NiiVue, which is built using web technologies. MRIcron is a desktop application, and therefore is unable to fill the nascent cloud and edge-based niches. Indeed, while tools like neurodesk (Renton et al., 2024) and brainlife (Hayashi et al., 2024) have allowed traditional desktop-based neuroimaging pipelines to scale to the cloud, there is a clear need for cloud-native visualization tools. Unlike my previous work, NiiVue has benefited from the collective wisdom of many developers and is built using the popular JavaScript (with low-level graphics using WebGL2 and the ability to use WebAssembly plug-ins). NiiVue is already providing visualization for ezBIDS (Levitas et al., 2024), TractoScope (Kruper et al., 2024), QSMxT (Stewart et al., 2022), OpenNeuro 38, FreeSurfer’s FreeBrowse, and AFNI’s QA tools (Taylor et al., 2024). Web technologies can also provide edge-based computing, where computations are done on the user’s own computer without requiring cloud resources. This can provide small applets for any device that can load a web page - with NiiVue already used in brain2print.org, niimath (Rorden et al., 2024), and brainchop (Plis et al., 2024). We are actively developing a wrapper for Jupyter notebooks (ipyniivue) providing Python developers with a comprehensive visualization tool for neuroimaging formats.

MRIcron’s drawing tools for creating lesion maps and other regions of interest are also time consuming and subjective. For example, de Haan (de Haan et al., 2015) and colleagues estimated the typical time for drawing the extent of a stroke was 18.4–25.1 min depending on modality. In contrast, their semi-automated method proved dramatically faster. Likewise, ITK-SNAP introduced semi-automated voxel drawing (Yushkevich et al., 2006). More recently, MRIcron (Liew et al., 2018) and ITK-SNAP (Liew et al., 2022) have been used to laboriously hand draw lesions for large public datasets from individuals with chronic stroke injury that can be used for training fully automated lesion mapping (Liew et al., 2022). Likewise, shared and curated acute injury datasets (Liu et al., 2023) have aided the development of fully automated lesion detection and lesion mapping (Liu et al., 2021). The detectability and extent of lesions depend on the time since injury as well as the modality (Forkel and Catani, 2018), so there is a continued need for diverse training datasets and methods for visualizing and correcting errors. These fully automated methods can provide an objective and scalable solution for the large datasets required to identify subtle effects or patterns of comorbidity.

Though MRIcron was initially developed to explore lesion-symptom relationships—a focus of my career—it has become widely popular as a versatile image processing tool. It is now frequently used with imaging data from healthy adults, studies on brain development in children, and research involving other animals. Interestingly, this aligns with John Duncan’s vision that converging evidence from multiple tools is essential for advancing our understanding of the brain.

In summary, MRIcron has played a pivotal role in the neuroimaging community by providing a free, open-source, and cross-platform tool that addresses many of the visualization and analysis needs of researchers and clinicians. MRIcron’s ability to handle a wide range of neuroimaging formats and its support for voxel-based lesion-symptom mapping have made it a valuable resource in the study of brain function and injury. However, as the field of neuroimaging continues to evolve, so too must the tools that support it.

This manuscript serves as a long-overdue documentation of MRIcron’s contributions and limitations, as well as a reflection on the visionary influence of John Duncan in the development of neuroimaging infrastructure. As we move forward, it is important to continue building on this foundation, ensuring that future tools not only meet the immediate needs of the community but also anticipate and adapt to the challenges and opportunities that lie ahead.

## Data Availability

No data was used for the research described in the article.
